# Cosmetic Acupuncture for Facial Aging: Anatomy, Mechanobiology, and Clinical Evidence—A Narrative Review

**DOI:** 10.1111/jocd.71245

**Published:** 2026-10-16

**Authors:** Magdalena Grzonkowska, Jagoda Baumgart, Michał Kułakowski, Mariusz Baumgart

**Affiliations:** ^1^ Department of Normal Anatomy The Ludwik Rydygier Collegium Medicum in Bydgoszcz, the Nicolaus Copernicus University in Toruń Toruń Poland; ^2^ Clinical Department of Orthopedics and Traumatology Jan Biziel University Hospital No. 2 in Bydgoszcz, Nicolaus Copernicus University in Toruń Toruń Poland

**Keywords:** cosmetic acupuncture, extracellular matrix, facial aging, facial anatomy, fibroblasts, mechanotransduction, skin rejuvenation

## Abstract

**Background:**

Cosmetic acupuncture is increasingly used for facial rejuvenation, but many proposed mechanisms are extrapolated from general acupuncture research, skin mechanobiology, wound‐healing models, or related percutaneous procedures. Direct evidence linking facial needling to structural remodeling of human skin remains limited. The aim of this review was to examine the anatomical and mechanobiological basis of cosmetic acupuncture and distinguish demonstrated effects from plausible but unverified mechanisms.

**Methods:**

MEDLINE/PubMed and ClinicalTrials.gov were searched through 14 September 2026, supplemented by citation tracking and targeted journal and publisher searches. Clinical studies were integrated with evidence from needle biomechanics, fibroblast mechanotransduction, extracellular matrix biology, neurovascular and immune signaling, neuromuscular physiology, and facial anatomy. Evidence was interpreted according to its directness to cosmetic facial needling.

**Results:**

Acupuncture‐related studies demonstrate connective‐tissue deformation, fibroblast cytoskeletal responses, local purinergic and vascular effects, and context‐dependent neuromuscular changes, although much of this evidence is nonfacial or experimental. Clinical studies suggest improvement in selected wrinkles, folds, and facial‐contour outcomes. Direct evidence of neocollagenesis, elastin remodeling, or persistent extracellular matrix regeneration in human facial skin after conventional filiform cosmetic acupuncture is lacking.

**Conclusions:**

Cosmetic acupuncture can be viewed as a tissue‐specific mechanical intervention rather than a uniform “rejuvenation” procedure. Depth, anatomical target, manipulation, puncture density, and treatment frequency may influence the biological response. Future studies should directly link defined mechanical exposure and tissue targeting to objective biological and clinical endpoints.

## 1 Introduction

Facial aging is a multilayered process involving the skin, superficial and deep adipose compartments, fibrous retaining structures, facial muscles, and the skeleton [1, 2, 3, 4]. The visible phenotype therefore does not arise from the dermis alone. Within aged skin, fragmentation of the collagenous extracellular matrix (ECM) reduces mechanical support for resident fibroblasts and is associated with impaired cell spreading, altered transforming growth factor‐β signaling, reduced collagen production, and increased matrix‐degrading activity [5, 6, 7, 8]. This mechanical dimension of dermal aging is relevant to procedures whose primary input is physical rather than pharmacological.

Cosmetic acupuncture, also described as facial or facial rejuvenation acupuncture, comprises heterogeneous techniques intended to modify wrinkles, folds, skin appearance, or facial contour [9, 10, 11, 12, 13, 14, 15, 16]. Published protocols differ markedly in needle number, diameter, depth, trajectory, manipulation, retention, treatment frequency, and use of distal points (Figure 1). Some reports additionally include needle‐knife procedures or electrical stimulation that are not mechanically equivalent to conventional filiform needling [12, 16]. Consequently, “cosmetic acupuncture” should not be treated as a single biological intervention.

**FIGURE 1:**
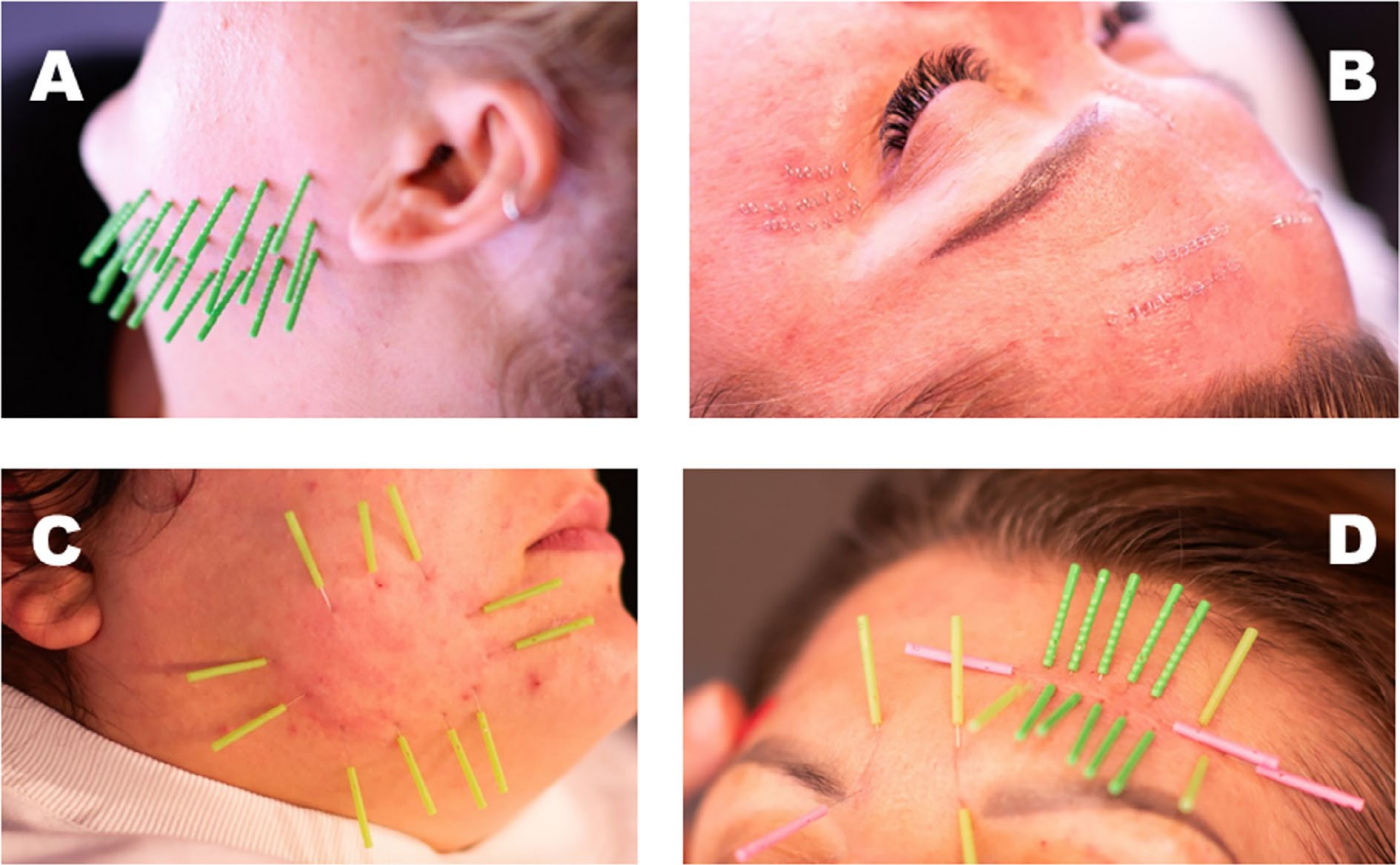
Examples of the diversity of needling applications used in cosmetic acupuncture: (A) short 7‐mm needles inserted at approximately 45°; (B) 3‐mm intradermal needles inserted along wrinkle lines; (C) long 30‐mm needles inserted at a shallow angle of approximately 5°; and (D) 7‐ and 15‐mm needles inserted around a wrinkle.

The mechanistic literature is similarly heterogeneous. Needle rotation can increase needle–tissue coupling, wind connective tissue around the needle, produce tissue displacement beyond the puncture tract, and induce fibroblast cytoskeletal remodeling [17, 18, 19, 20, 21, 22, 23, 24, 25]. Acupuncture can also produce local purinergic and vascular responses [26, 27, 28, 29] and alter masticatory‐muscle activity or morphology under selected conditions [13, 30, 31, 32]. Because most of these observations were obtained outside cosmetic facial acupuncture, they support biological plausibility but do not establish collagen synthesis or persistent structural rejuvenation of facial skin. This review therefore distinguishes direct cosmetic‐acupuncture evidence from indirect mechanistic support.

## 2 Review Scope and Evidence Framework

The original literature search was completed on 11 August 2026 and was updated on 14 September 2026 during revision of the manuscript. MEDLINE/PubMed was used as the principal biomedical source, and ClinicalTrials.gov was searched for ongoing or completed studies. Searches were supplemented by backward and forward citation tracking of relevant publications and targeted searches of journal and publisher databases. No publication‐date restriction was imposed. The review was structured according to SANRA principles for narrative reviews [33], while PRISMA‐S informed the transparent reporting of the search strategy without implying systematic‐review status [34]. STRICTA and ACURATE informed interpretation of acupuncture and sham‐intervention reporting [35, 36].

Publications were selected if they addressed either (1) clinical outcomes after cosmetic or facial acupuncture or (2) one of the prespecified mechanistic domains. For clinical outcomes, priority was given to original human studies; for mechanistic claims, to original studies directly measuring the relevant biomechanical or biological process. Reviews were used mainly for context and source identification. Evidence from animal, nonfacial, wound‐healing, microneedling, or dry‐needling models was retained only to address a mechanistic gap and was explicitly classified as indirect or analogical. Thread embedding, needle‐knife procedures, transcutaneous electrical acupoint stimulation, and auricular acupuncture were considered separately because they do not provide mechanical exposure equivalent to conventional facial filiform needling.

Evidence was organized into four levels: direct human cosmetic‐acupuncture evidence; mechanistic acupuncture evidence from other tissues or settings; supportive facial‐anatomical or mechanobiological evidence; and analogical evidence from related procedures. Acute physiological effects were distinguished from delayed structural remodeling. Because this was a structured narrative synthesis, no meta‐analysis, study‐flow count, or single risk‐of‐bias instrument was applied across the heterogeneous evidence types. Methodological limitations were assessed qualitatively, including controls, blinding, sample size, outcome objectivity, follow‐up, intervention reporting, and the degree of extrapolation required.

## 3 Anatomy and Mechanical Dose

Facial tissues vary substantially by region. Skin thickness, superficial and deep fat architecture, retinacular structures, facial spaces, muscle depth, and neurovascular anatomy all influence what a needle encounters along its trajectory [1, 2, 3, 4, 37, 38, 39, 40, 41, 42]. An intradermal insertion therefore constitutes a different intervention from an oblique subcutaneous trajectory or an intramuscular insertion. Depth is not simply a marker of treatment intensity; it changes the target tissue and the plausible mechanism.

For conventional filiform needling, the initial event is mechanical. The practitioner controls needle diameter, inserted length, angle, number, and density of punctures, manipulation, and treatment repetition. For the purposes of this review, these variables are grouped conceptually as the applied mechanical dose; this is a descriptive framework rather than a validated dosimetric measure. The stimulus actually received by tissue will additionally depend on regional thickness, stiffness, hydration, collagen organization, fascial architecture, and muscle morphology. Thus, identical surface protocols need not generate identical tissue responses.

Among directly measured needle–tissue phenomena, needle grasp provides comparatively strong evidence of mechanical coupling. Human and experimental studies show increased withdrawal force with rotation, connective‐tissue winding, and collagen involvement in needle coupling [17, 18, 19, 20]. Ultrasound elastography further demonstrates tissue displacement beyond the immediate needle tract and anisotropic motion related to connective‐tissue organization [21, 22]. These studies were performed predominantly outside facial cosmetic settings, so transfer of their spatial findings to facial dermis remains inferential. Whether comparable mechanical fields occur in facial tissues, and how far they extend laterally, has not been established.

This uncertainty is central to the question of needle number. No study defines a minimum or optimal number of needles for facial rejuvenation, and needle count alone is an inadequate surrogate for biological dose. A small number of needles cannot be assumed to activate fibroblasts throughout the face; conversely, more needles cannot be assumed to produce more collagen. For dermal protocols, puncture density, depth, tissue target, and manipulation may ultimately prove more informative than total needle number, but this has not been tested directly.

## 4 Fibroblast Mechanotransduction and Extracellular Matrix Remodeling

Fibroblasts are mechanosensitive cells. In aged human dermis, loss of normal collagen support alters cell shape and mechanical force and is linked to dysregulated collagen homeostasis [5, 6, 7, 8]. Acupuncture‐related experiments provide direct evidence that acupuncture‐associated mechanical loading can alter fibroblast morphology and cytoskeletal organization: connective‐tissue stretch induces fibroblast spreading and remodeling, and the response varies with the amount of bidirectional needle rotation [23, 24, 25]. These experiments were conducted largely in nonfacial experimental connective tissue and therefore support mechanistic plausibility for facial needling rather than demonstrating the same response in human facial dermis.

The downstream molecular pathways are less directly established. Focal adhesion signaling and actin organization regulate traction‐dependent matrix remodeling and TGF‐β receptor function in fibroblasts [43, 44]. YAP/TAZ participate in mechanosensitive regulation of dermal fibroblast function [45, 46], while PIEZO1 provides another plausible interface between mechanical loading and fibroblast activation [47, 48, 49]. Together, these findings provide a biologically coherent framework for mechanotransduction, but these pathways have not been measured after cosmetic acupuncture in human facial skin and should therefore be regarded as candidate downstream mechanisms.

Fibroblast activation is not synonymous with collagen synthesis. Mechanical stimulation can alter proliferation, migration, contractility, cytokine production, matrix synthesis, and matrix degradation, and the direction and magnitude of the response depend on dose and tissue context. The available data therefore support a testable hypothesis of a non‐linear dose–response relationship rather than an established “mechanobiological therapeutic window” for cosmetic acupuncture [24, 46, 47, 48, 49, 50].

Wound‐healing studies provide additional but indirect evidence. In animal injury models, acupuncture has influenced fibroblast proliferation, angiogenesis, and macrophage‐associated repair pathways [51, 52]. These models cannot be equated with intact facial skin. Microneedling offers stronger human evidence that sufficiently dense percutaneous injury can induce collagen‐related dermal remodeling [53, 54, 55], but its puncture density and injury pattern differ substantially from conventional acupuncture. Microneedling therefore demonstrates biological possibility, not procedural equivalence.

In the literature identified in this review, no human study of conventional filiform cosmetic acupuncture directly demonstrated increased Type I or III collagen, procollagen, elastin, or histological neocollagenesis in facial skin. The evidence therefore supports a narrower conclusion: cosmetic acupuncture may generate mechanical and cellular signals capable of modifying fibroblast‐ECM interactions, whereas persistent structural remodeling and neocollagenesis in human facial skin remain unverified.

## 5 Purinergic, Neurovascular, Immune, and Neuromuscular Pathways

Needling also elicits local biochemical responses. Experimental work implicates extracellular purines, and human microdialysis has demonstrated increased interstitial adenosine after acupuncture [26, 27]. Human studies also show acute changes in skin or muscle blood flow, with nitric oxide contributing to cutaneous vasodilation [28, 29]. These findings support acute local purinergic and perfusion effects, but not persistent microvascular remodeling, angiogenesis, or a demonstrated mechanism for long‐term aesthetic change.

Sensory and immune pathways are plausible components of the same local response. CGRP has been linked to acupuncture‐related changes in blood flow, while animal studies describe interactions involving cutaneous nerve terminals and mast cells [56, 57, 58]. Their relevance to cosmetic facial outcomes remains inferential because direct evidence in intact human facial skin is lacking.

When needles reach facial or masticatory muscles, neuromuscular mechanisms become relevant. Reduced electrical activity, reduced resting tone, reduced stiffness, relaxation, volume loss, weakness, and atrophy are distinct phenomena. EMG studies in masticatory muscles show context‐dependent changes after acupuncture [30, 31]. In a randomized trial comparing acupuncture with botulinum toxin for masticatory myofascial pain, clinical improvement occurred with acupuncture, whereas marked EMG suppression was associated primarily with botulinum toxin [32]. These data argue against describing acupuncture as a mild form of chemodenervation, and findings from masticatory muscles should not be assumed to generalize directly to individual mimetic muscles.

Ogino et al. reported an approximately 7% reduction in MRI‐derived masseter volume after eight weekly facial‐acupuncture sessions, but the study included only 10 women and lacked a control group, EMG, bite‐force testing, or long‐term imaging [13]. Similarly, improvement in glabellar lines during maximum frowning in the randomized trial by Haghir et al. is compatible with a neuromuscular contribution, but muscle physiology was not measured [14]. “Neuromuscular modulation” is consequently more accurate than generalized “muscle relaxation.” These findings support a mechanistic hypothesis rather than a demonstrated causal explanation for wrinkle or contour improvement. An evidence‐weighted conceptual model integrating anatomical target, mechanical input, biological pathways, temporal sequence, and degree of mechanistic certainty is presented in Figure 2.

**FIGURE 2:**
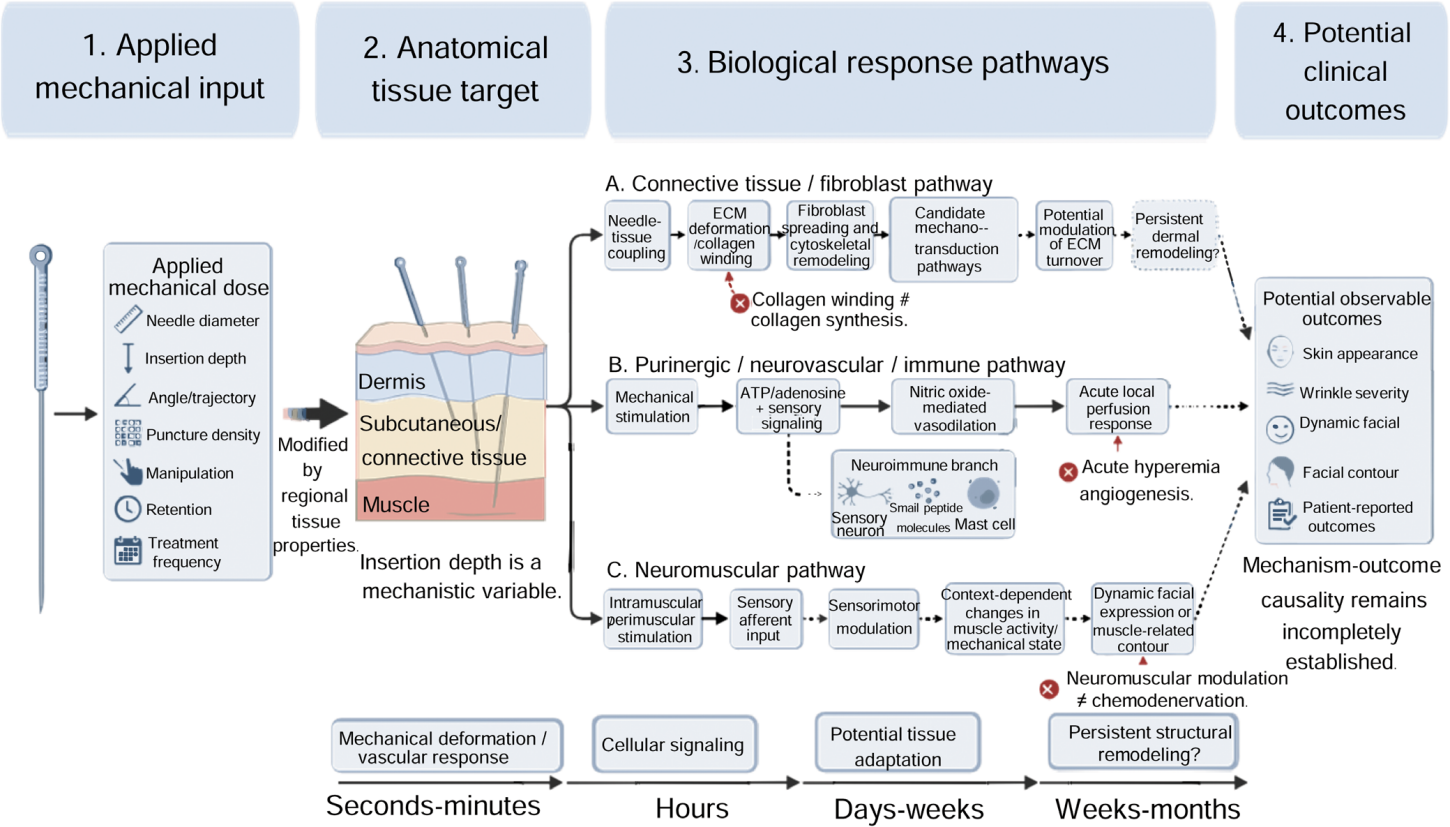
Evidence‐weighted mechanistic model of cosmetic acupuncture.

## 6 Clinical Evidence and Mechanistic Interpretation

The clinical literature remains small and heterogeneous. The 2018 systematic review identified seven prospective studies involving 216 participants and found insufficient evidence for firm conclusions [9]. Subsequent studies have reported changes in facial topography or elasticity‐related measures [10], periocular wrinkles and nasolabial folds [11, 12, 13, 14, 15], masseter morphology [13], and patient‐reported or photographic outcomes [14, 15, 16]. Preliminary studies have also described changes in skin moisture or sebum after facial needling [59] and improvement after combined stuck and facial acupuncture [60]. A 2026 retrospective JCD study of 217 cases reported improvement after auricular acupuncture for facial aesthetics [61]; because facial tissues were not needled, those findings cannot support a local facial mechanotransduction mechanism.

The strongest recent direct clinical evidence includes the wait‐list‐controlled glabellar‐line trial by Haghir et al. [14], the MRI masseter study [13], and the quasi‐experimental nasolabial‐fold study by Nurmawati et al. [15]. Each provides a clinical signal, but none directly links the observed outcome to collagen synthesis, a specific fibroblast pathway, or measured facial‐muscle inhibition. Luo et al. compared transcutaneous electrical acupoint stimulation, acupuncture, and sham TEAS in facial aging [16], but sham TEAS is not a sham control for penetrating acupuncture, and electrical stimulation constitutes a different biological input.

Across the clinical literature, outcome and mechanistic measurements remain largely disconnected. Studies reporting cosmetic improvement have not simultaneously quantified tissue deformation, fibroblast signaling, collagen or elastin synthesis, adenosine‐mediated perfusion, or facial‐muscle physiology. Mechanistic evidence from nonfacial acupuncture, cell and tissue models, and related percutaneous procedures should therefore be used to generate hypotheses rather than assign causality. Thus, wrinkle improvement does not demonstrate neocollagenesis, reduced masseter volume does not establish muscle relaxation, and immediate changes in appearance are more compatible with acute physiological effects than with newly synthesized collagen. Table 1 summarizes the directness of the evidence and the extent to which each proposed mechanism can currently be linked to cosmetic outcomes.

**TABLE 1:** Evidence‐weighted interpretation of proposed mechanisms of cosmetic acupuncture and their linkage to clinical outcomes.

Domain	Representative evidence	Directness to cosmetic acupuncture	Current interpretation and clinical linkage
Needle‐tissue mechanics	Withdrawal force, connective‐tissue winding, tissue displacement [17, 18, 19, 20, 21, 22]	Direct acupuncture biomechanics, predominantly nonfacial; facial spatial extent not defined	Directly demonstrated at the tissue‐mechanics level. A causal link to cosmetic outcome has not been established.
Fibroblast response	Spreading and cytoskeletal remodeling after acupuncture‐associated stretch [23, 24, 25]	Direct mechanistic evidence in experimental/nonfacial connective tissue; not measured in human facial dermis	Directly demonstrated as a fibroblast response, but not equivalent to collagen synthesis and not yet linked to clinical facial improvement.
YAP/TAZ, PIEZO1, TGF‐β/FAK	Dermal and fibroblast mechanobiology [43, 44, 45, 46, 47, 48, 49, 50]	Supportive mechanobiology; no direct measurement after cosmetic facial acupuncture	Biologically plausible candidate pathways. Their involvement in cosmetic‐acupuncture outcomes remains unverified.
ECM remodeling	Human microneedling histology [53, 54, 55]	Analogical evidence from a procedure with different puncture density and injury pattern	Demonstrates biological possibility, not procedural equivalence; neocollagenesis after cosmetic acupuncture remains unverified.
Purinergic/vascular	Human adenosine and perfusion studies [27, 28, 29]	Direct acupuncture evidence, largely nonfacial and focused on acute physiology	Acute effects are directly supported, but persistent vascular remodeling and their contribution to cosmetic outcomes are not established.
Neuromuscular	EMG studies, masseter MRI, glabellar clinical outcomes [13, 14, 30, 31, 32]	Mixed physiological and clinical evidence, mainly masticatory; direct mimetic‐muscle mechanisms are unmeasured	Neuromuscular involvement is plausible and partly supported, but specific causal pathways linking muscle effects to cosmetic outcomes remain incomplete.

## 7 Clinical Implications, Safety, and Research Priorities

The mechanistic synthesis suggests that tissue target should be reported alongside traditional point selection rather than assuming that all facial needle placements represent the same biological exposure. A superficial dermal protocol, a subcutaneous oblique technique, and intramuscular needling should therefore be described as technically and anatomically distinct interventions. Future studies should specify depth, trajectory, needle diameter, puncture density, manipulation, retention, frequency, and intended tissue layer, extending current STRICTA reporting [35].

Safety must be considered within the same dose framework. Large prospective acupuncture cohorts indicate that most adverse events are mild and transient, but bleeding, bruising, pain, infection, and rare serious complications are documented [62, 63, 64, 65]. Facial risk also changes with depth because the glabellar, periorbital, nasolabial, and midfacial regions contain variable neurovascular structures [66, 67, 68, 69]. Greater depth or manipulation should therefore not be justified simply on the assumption that a stronger stimulus produces a better regenerative effect.

The next research step is not simply a larger uncontrolled efficacy study. Priority should be given to mechanism‐linked trials that quantify tissue exposure and verify the proposed target. High‐frequency ultrasound and elastography could define insertion depth and local deformation; dermal studies could directly assess fibroblast morphology, YAP/TAZ, PIEZO1, TGF‐β signaling, procollagen, collagen I/III, and MMP/TIMP balance, and neuromuscular trials could combine standardized dynamic imaging with EMG, ultrasound, or elastography. Dose–response studies should independently vary puncture density, depth, and manipulation. The critical translational sequence is a defined intervention, confirmed tissue target, measured biological response, persistent structural adaptation, and clinically meaningful outcome; each link should be measured rather than inferred from the next.

## 8 Conclusions

Cosmetic acupuncture is best conceptualized as a tissue‐specific mechanical intervention whose biological effects depend on the tissue reached and the mechanical exposure delivered. Evidence is strongest for needle–tissue coupling, tissue deformation, fibroblast cytoskeletal remodeling, acute purinergic and vascular responses, and selected neuromuscular changes. However, the clinical and mechanistic literatures remain incompletely linked.

Direct activation of YAP/TAZ, PIEZO1, or TGF‐β/SMAD by cosmetic acupuncture in human facial skin, as well as histological neocollagenesis or elastin remodeling, has not been demonstrated. Future studies should therefore define the tissue target and mechanical exposure and measure the predicted biological response alongside objective clinical outcomes. This approach may allow cosmetic acupuncture to be evaluated as a reproducible, dose‐characterized intervention while preserving a clear distinction between biological plausibility and demonstrated mechanism.

## Conflicts of Interest

The authors declare no conflicts of interest.

## Data Availability

Data sharing not applicable to this article as no datasets were generated or analysed during the current study.
